# Correction: A new mechanism of arterial calcification in diabetes: interaction between H3K18 lactylation and CHI3L1

**DOI:** 10.1042/CS20243122_COR

**Published:** 2026-05-06

**Authors:** Yi Zhu, Jing-cheng Chen, Jia-li Zhang, Fang-fang Wang, Rui-ping Liu

**Affiliations:** 1 Department of Cardiology, The Affiliated Changzhou Second People’s Hospital of Nanjing Medical University, Changzhou Second People’s Hospital, Changzhou Medical Center, Nanjing Medical University, Changzhou 213000, P.R, China; 2 Department of Gastrointestinal Surgery, The Affiliated Changzhou Second People’s Hospital of Nanjing Medical University, Changzhou 213000, P.R, China; 3 Department of Orthopaedics, The Affiliated Changzhou Second People’s Hospital of Nanjing Medical University, Changzhou, 213000, P.R, China

**Keywords:** CHI3L1, diabetic arterial calcification, H3K18la, lactylation, VSMC

The authors of the original article “A new mechanism of arterial calcification in diabetes: interaction between H3K18 lactylation and CHI3L1” (DOI: 10.1042/CS20243122) would like to correct Figure 6A. Due to an error in the preparation of Figure 6A, an incorrect image was inadvertently used for the CHI3L1 WT(+/+), DM + VDN panel. The authors contacted the Journal regarding the error, along with an explanation and a replacement figure containing the correct data. The requested correction has been assessed and agreed to by the Editorial Board. The authors declare that these corrections do not change the results or conclusions of their paper.

The corrected version of Figure 6A is presented here.

**Figure 6:**
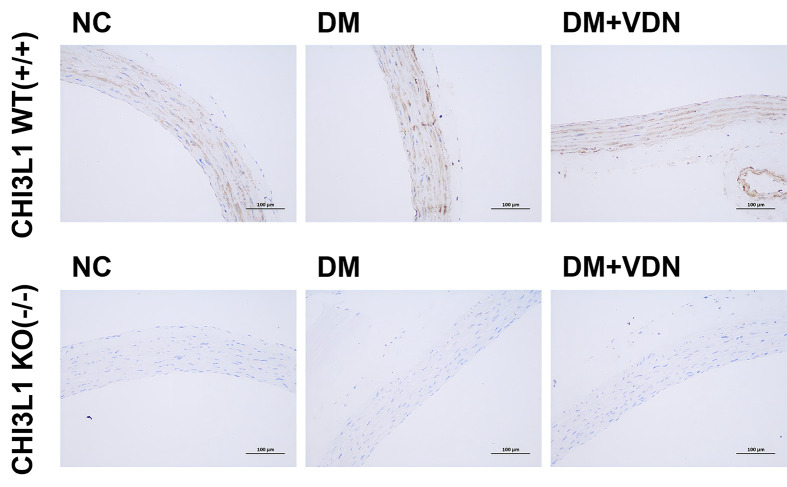
CHI3L1 knockout reduced arterial calcification in diabetes We constructed a CHI3L1 whole gene knockout mouse and prepared a mouse model of diabetes or diabetic arterial calcification. **(A)** Immunohistochemical staining of CHI3L1 in the arterial tissues of CHI3L1 non-knockout mice and CHI3L1 knockout mice in the normal state, diabetic model and diabetic artery calcification model; at least 6 repeats per condition were analyzed.

